# Posttranslational protein modifications as gatekeepers of cancer immunogenicity

**DOI:** 10.1172/JCI180914

**Published:** 2024-05-15

**Authors:** Emanuela Marchese, Shadmehr Demehri

**Affiliations:** 1 Center for Cancer Immunology, Krantz Family Center for Cancer Research, and; 2 Cutaneous Biology Research Center, Department of Dermatology, Massachusetts General Hospital and Harvard Medical School, Boston, Massachusetts, USA.

## Abstract

Triple-negative breast cancer (TNBC) presents a formidable challenge in oncology due to its aggressive phenotype and the immunosuppressive nature of its tumor microenvironment (TME). In this issue of the *JCI,* Zhu, Banerjee, and colleagues investigated the potential of targeting the OTU domain-containing protein 4 (OTUD4)/CD73 axis to mitigate immunosuppression in TNBC. They identified elevated CD73 expression as a hallmark of immunosuppression in TNBC. Notably, the CD73 expression was regulated by OTUD4-mediated posttranslational modifications. Using ST80, a pharmacologic inhibitor of OTUD4, the authors demonstrated the restoration of cytotoxic T cell function and enhanced efficacy of anti-PD-L1 therapy in preclinical models. These findings underscore the therapeutic potential of targeting the OTUD4/CD73 axis in TNBC.

## Overcoming immunosuppressive tumor microenvironment in breast cancer

Triple-negative breast cancer (TNBC) represents a subtype of breast cancer characterized by the absence of estrogen receptor (ER), progesterone receptor (PR), and human epidermal growth factor receptor 2 (HER2) expression (1). Despite advancements in cancer treatment, TNBC remains a formidable challenge due to its aggressive nature, high rates of recurrence, and limited targeted therapeutic options (2). A key feature contributing to the aggressive behavior of TNBC is its ability to form an immunosuppressive, “cold” tumor microenvironment (TME). This immunosuppressive TME poses substantial limitations to the efficacy of immunotherapy in patients with TNBC. Understanding the molecular and cellular mechanisms underlying the cold TME phenotype in TNBC is essential for developing immunotherapeutic approaches to overcome these limitations and improve patient outcomes.

In this issue of the *JCI*, Zhu, Banerjee, and colleagues unveil the potential of the OTU domain-containing protein 4 (OTUD4)/CD73 proteolytic axis as a promising target for bolstering the immunogenicity of TNBC and enhancing the effectiveness of immunotherapy in a complex TME context (3). The research approach employed multi-omic analyses to investigate the underlying mechanism of the immunosuppressive TME observed in TNBC. Through these efforts, the authors identified a distinct immunosuppressive signature within a subset of TNBCs characterized by elevated expression of CD73 (3).

CD73 exerts its immunosuppressive effect on various immune cells, including T cells, B cells, dendritic cells, and natural killer cells, by catalyzing the conversion of extracellular adenosine monophosphate (AMP) to inhibitory adenosine (4). Maintaining the equilibrium between ATP and adenosine is essential to prevent unchecked tissue damage from excessive inflammatory reactions (5). Thus, CD73, a rate-limiting enzyme for adenosine production, is crucial in preserving tissue homeostasis and regulating the delicate balance necessary for immune activation and tissue protection.

CD73 expression in breast cancer cells is regulated via various factors, such as HIF-α and the estrogen receptor (ER), primarily at the transcriptional level. Zhu, Banerjee, and colleagues spotlight the potential impact of CD73’s aberrant posttranslational modifications on the TNBC’s cold TME phenotype (3). These findings are reminiscent of other immune checkpoint proteins, such as PD-L1, in which posttranslational modifications have a pivotal role in their regulation (6). CD73 regulation entails a sophisticated process involving E3 ligase TRIM21-mediated destruction and OTUD4-catalyzed stabilization. This intricate regulatory mechanism governs CD73 abundance on the cell membrane, whereby heightened CD73 deubiquitylation by OTUD4 augments its functionality.

In exploring the potential association between increased CD73 expression and reduced immunogenicity in a subset of TNBC, the authors conducted a Cybersort analysis. This analysis revealed a correlation between elevated CD73 expression in breast tumors and decreased infiltration of CD8^+^ T cells. By examining 1,080 TNBC samples from The Cancer Genome Atlas (TGCA) database, the authors showed improvement in patient survival among those with breast tumors exhibiting high CD8^+^ T cell infiltration and low levels of CD73 expression. The findings determine a notable correlation between the aberrant increase in CD73 expression and the poor prognosis of immune-cold TNBCs (3).

CD73 is expressed in various solid tumors in humans, and its presence often correlates with unfavorable cancer progression, including metastasis and angiogenesis (7). Its immunosuppressive effects encompass inhibition of T cell activation by disrupting T cell receptor signaling and cytokine production, induction of regulatory T cells, suppression of proinflammatory cytokine production, and, in some instances, inhibition of antigen-presenting cells (8). These effects primarily stem from CD73’s enzymatic activity. Beyond immune modulation, CD73 can influence diverse aspects of tumorigenesis, such as adhesion, migration, invasion, and the stemness of cancer cells (7). Additionally, a soluble form of CD73 (sCD73) was found at elevated levels in the serum of patients with cancer compared with individuals who were healthy (9). However, the precise role of sCD73 remains poorly understood.

CD73 expression on cancer cells has been associated with increased resistance to various therapies, including chemotherapy, radiation therapy, and immunotherapy. In TNBC, CD73 expression is associated with doxorubicin resistance (10); tumor cells treated with doxorubicin increase CD73 expression, leading to CD8^+^ T cell suppression. Further, increased CD73 levels in TNBC have been reported following exposure to other chemotherapeutic agents like carboplatin and gemcitabine (11). Upregulated CD73 after chemotherapy likely serves as a compensatory mechanism aimed at counteracting the surplus ATP released from dying tumor cells after therapy (12).

OTUD4 is a member of the ovarian tumor–associated proteases domain-containing proteins (OTUDs) family, functioning as a deubiquitinase. It interacts with myeloid differentiation primary response 88 (MyD88) and mitochondrial antiviral-signaling protein (MAVS), which are pivotal adaptor proteins involved in innate immune signaling pathways activated by viral infections (13). Through these interactions, OTUD4 potentially disrupts downstream signaling cascades crucial for eliciting antiviral immune responses. Additionally, studies suggest that OTUD4 plays a role in modulating toll-like receptors (TLRs), which are essential for recognizing viral pathogens and initiating immune reactions. Consequently, OTUD4 dysregulation may hinder cells’ ability to detect and mount responses against viral infections via TLR-mediated pathways (14).

Moreover, OTUD4 is known to participate in DNA alkylation damage repair, indicating a potential role in malignancy. However, its involvement in cancer remains unclear, and research specifically investigating the role of OTUD4 in cancer immunology is limited. Notably, recent findings have shown that OTUD4 is overexpressed in glioblastoma, a highly aggressive primary malignant brain tumor. In this context, OTUD4 plays a crucial role in cell proliferation, invasion, and clonogenic capacity by deubiquitinating CDK1 and activating the MAPK signaling pathway (15).

## Mechanistic insight into CD73 regulation

**Figure 1:**
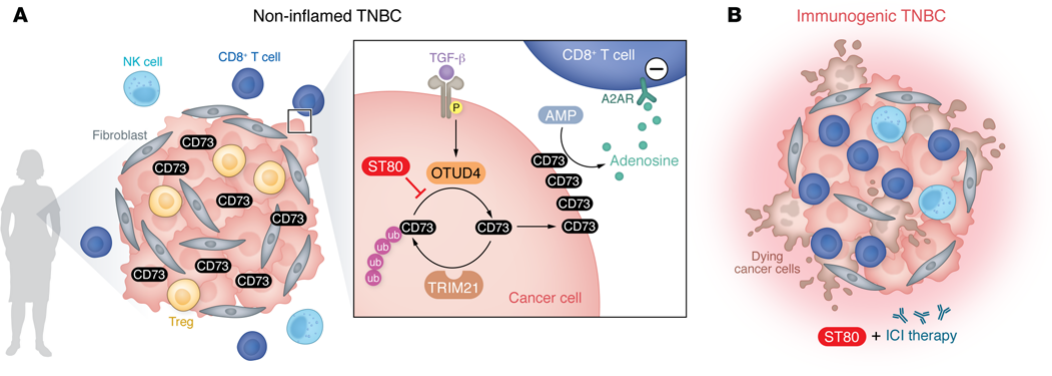
Targeting the OTUD4/CD73 proteolytic axis enhances immunogenicity in TNBC. (**A**) Posttranscriptional regulation of CD73 involves TRIM21-mediated destruction and OTUD4-catalyzed stabilization. Zhu, Banerjee, and colleagues demonstrated that elevated TGF-β levels, commonly found in an immunosuppressive TME, activate OTUD4, leading to deubiquitylation of CD73. Stabilized CD73 on the cancer cell membrane promotes immune evasion by increasing adenosine levels in the TME, which suppresses CD8^+^ T cells. The inhibitor ST80 specifically disrupts the proteolytic interaction between CD73 and OTUD4. (**B**) The presence of ST80 reinvigorates cytotoxic CD8^+^ T cells, enhancing TNBC immunogenicity. Thus, ST80 may increase the efficacy of immune checkpoint inhibitor (ICI) therapy for TNBC.

Zhu, Banerjee, and colleagues used mass spectrometry to explore the CD73 interactome. Subsequent immunoprecipitation assays consistently showed CD73 interacting with OTUD4 in TNBC cells, and in situ detection techniques showed the interaction occurred within the cytosol. These findings suggest OTUD4 may drive CD73 accumulation in TNBC (3). Notably, OTUD4 overexpression heightened levels of membrane-bound CD73 in cancer cells. OTUD4-mediated stabilization of CD73 suppressed cytotoxic CD8^+^ T cell function, thereby facilitating tumor immune destruction. In contrast, OTUD4 knockdown resulted in increased CD73 ubiquitylation and subsequent degradation, and enhanced T cell proliferation and IFN-γ production. These observations highlight the role of the deubiquitinase OTUD4 in regulating CD73 protein levels that affect extracellular adenosine production and T cell dysfunction within the TNBC TME. Mechanistically, transforming growth factor-β (TGF-β) signaling orchestrated the OTUD4/CD73 proteolytic axis, promoting tumor progression (3). The upregulation of TGF-β (commonly found in a tumor microenvironment) coopted OTUD4 to deubiquitylate CD73 hyperactively, potentiating its stability and membrane abundance, immune evasion, and maintaining tumor progression (Figure 1) (3).

## Therapeutic potential and clinical relevance

An important therapeutic advance proposed by Zhu, Banerjee, and colleagues is the development of ST80, a pharmacologic inhibitor designed to target the physical interaction between OTUD4 and CD73 (Figure 1) (3). This inhibitor disrupted the interaction between OTUD4 and CD73, leading to the ubiquitylation and subsequent degradation of CD73. The coculture of breast cancer cells with human PBMC after ST80 treatment led to more pronounced cytotoxicity. ST80 treatment also increased cytotoxicity against a broad range of cancer cell lines, including human non-small cell lung cancer (HCC827), ovarian cancer (SKOV3), and colon cancer (HCT116) (3).

In recent years, CD73 inhibitors have been developed and are presently under evaluation in clinical trials. These inhibitors are broadly divided into small molecule compounds (further categorized into nucleotide-based and nonnucleotide-based inhibitors) and monoclonal antibodies, each with distinct advantages and drawbacks. Small molecule inhibitors offer the convenience of oral administration and improved distribution, facilitating better tumor penetration and tissue barrier traversal. However, they may display reduced specificity and potential off-target effects due to binding to conserved sites across various enzymes. Conversely, monoclonal antibodies offer heightened specificity and lower toxicity but can be costly, with limited tissue penetration as a drawback.

Among nucleotide-based small molecules, AB680 is a promising CD73 inhibitor capable of reversing adenosine-mediated inhibition of antitumor immune responses in preclinical models (16). Most monoclonal antibodies targeting CD73 act by blocking its catalytic domain, thereby inhibiting CD73 enzymatic function. Oleclumab exhibits a dual mechanism of action, inducing the crosslinking of CD73 dimers and impeding CD73 from adopting its catalytic form (17). One limitation of small molecules and antibodies targeting CD73 is their ability to reach intracellular compartments and block intracellular CD73 activity.

Notably, ST80 represents a compound capable of inhibiting the OTUD4/CD73 axis. Its therapeutic potential is further validated in orthotopic cancer models, where combining ST80 treatment with the FDA-approved anti–hPD-L1 antibody, durvalumab, demonstrated enhanced suppression of tumor growth and prolonged survival without evident toxic effects (3). ST80 treatment restored CD8^+^ T cell function, thereby enhancing tumor immunogenicity. This mechanism suggests that ST80 increases the sensitivity of TNBC to anti–PD-L1 therapy, even in cases of high OTUD4 and CD73 expression. Intriguingly, the depletion of CD8^+^ T cells abolished the efficacy of ST80, emphasizing the critical role of CD8^+^ T cells in ST80-mediated antitumor effects (3).

The reciprocal regulation of CD73 through ubiquitylation and deubiquitylation may represent a critical factor in controlling the immune response within the TME of breast and other cold tumors. Targeting the specific interaction between OTUD4 and CD73 offers a promising approach for TNBC treatment, especially in combination with immune checkpoint inhibitor therapy. Additionally, identifying a distinct immunosuppressive signature within a subset of TNBCs characterized by elevated expression of CD73 suggests the potential for personalized treatment strategies. Patients with TNBC tumors exhibiting high CD73 expression may benefit from targeted therapies that disrupt the OTUD4/CD73 axis.

## Next questions

The combination of ST80 with anti–PD-L1 therapy shows promise in preclinical models (3), however, the potential synergistic effects of ST80 with other immunotherapeutic agents, conventional chemotherapy, and the impact of OTUD4/CD73 axis blockade in other cancers remain to be explored. While Zhu, Banerjee, and colleagues elucidate the intricate interplay between OTUD4 and CD73 in the context of advanced TNBC, an intriguing topic for further exploration relates to the developmental role these molecules have in the early stages of TNBC. Specifically, how do alterations in the OTUD4/CD73 proteolytic axis contribute to the initiation and progression of TNBC from preinvasive to invasive stages? Given the emerging evidence implicating CD73 in tumor immune evasion, what is the impact of CD73 dysregulation on immune surveillance mechanisms during the early stages of TNBC? Finally, the long-term effects of targeting the OTUD4/CD73 axis in TNBC remains unknown, including the durability of treatment response, potential development of resistance mechanisms and adverse events, and the overall impact on patient survival. Answering these questions will pave the way to an in-depth understanding of the molecular mechanisms underlying TNBC pathogenesis and offer targets for early intervention strategies.

## Version 1 05/15/2024

Electronic publication
